# Inflammation and Depression: A Nervous Plea for Psychiatry to Not Become Immune to Interpretation

**DOI:** 10.3390/ph12010029

**Published:** 2019-02-14

**Authors:** Jan Pieter Konsman

**Affiliations:** Aquitaine Institute for Integrative and Cognitive Neuroscience (INCIA) UMR CNRS 5287, University of Bordeaux, 146 rue Léo Saignat, 33076 Bordeaux, France; jan-pieter.konsman@u-bordeaux.fr

**Keywords:** depression, inflammation, immunopsychiatry, interleukin, psychoneuroimmunology, stress

## Abstract

The possibility that inflammation plays a causal role in major depression is an important claim in the emerging field of immunopsychiatry and has generated hope for new treatments. The aims of the present review are first to provide some historical background and to consider the evidence in favor of the claim that inflammation is causally involved in major depression. The second part discusses some of the possibilities allowed for by the use of broad ‘umbrella’ concepts, such as inflammation and stress, in terms of proposing new working hypotheses and potential mechanisms. The third part reviews proposed biomarkers of inflammation and depression and the final part addresses how elements discussed in the preceding sections are used in immunopsychiatry. The ‘umbrella’ concepts of inflammation and stress, as well as insufficiently-met criteria based inferences and reverse inferences are being used to some extent in immunopsychiatry. The field is therefore encouraged to specify concepts and constructs, as well as to consider potential alternative interpretations and explanations for findings obtained. The hope is that pointing out some of the potential problems will allow for a clearer picture of immunopsychiatry’s current strengths and limitations and help the field mature.

## 1. An Introduction to Immunopsychiatry

“Studying the communication between the brain and the immune system, …, is a hot area in psychiatry and neuroscience research, and has led to the introduction of a new term to define the field: Immunopsychiatry [1]” [2] (p. 197). The term ‘immunopsychiatry’ was introduced almost four decades ago to indicate that antibody-mediated and cell-mediated immune responses may be involved in the pathogenesis of mental diseases [3]. A little later, the notion of neuroimmune system was introduced and proposed to contribute to mental disorder when subject to autoimmune disease [4]. However, more recently, an additional claim regarding neuroimmune interactions and systems has gained traction and revived immunopsychiatry. Indeed, the idea that inflammation plays a causal role in major depression and other mental disorders is now a major claim in immunopsychiatry and has generated hope for new (personalized) treatments of mental disorders, as well as for increased molecular understanding allowing for psychiatry to become more scientific [2,5,6,7].

The aims of the present review are first to provide some historical background and to consider the evidence in favor of the claim that inflammation is causally involved in major depression. To do so, the link between medical conditions and mental disorders as well the relationship between the fields of immunopsychiatry and that of psychoneuroimmunology will first be dealt with. The second part will discuss some of the possibilities allowed for by the use of broad ‘umbrella’ concepts, such as inflammation and stress, in terms of proposing new working hypotheses and potential mechanisms. The third part will review some proposed biomarkers of inflammation and depression and the final part will address how elements discussed in the preceding sections seem to be used in reasoning within immunopsychiatry.

### 1.1. Medical Conditions, Inflammation and Mood

At the end of the 19th century, William Osler when describing the category progressive septicemia during which “organisms enter the blood from some local septic focus” remarked that: “There may be early delirium or marked mental prostration and apathy” (William Osler, The principles and practice of medicine, 1892, p. 115). Almost a century later, Benjamin Hart coined the term “sickness behavior” to describe “the sleepy or depressed or inactive animal [that] is less motivated to move about …” when sick [8] (p. 129). This, along with evidence obtained around 1980 showing that mortality is increased when force-feeding animals that show reduced spontaneous food intake after bacterial infection [9], led to the idea that behavioral changes observed during infection are host responses that contribute to pathogen elimination.

Although sickness behavior can be considered adaptive in response to acute infection, the similarity between the behavioral consequences of inflammation and depressive(-like) symptoms in humans and animals has been pointed out in the mid-1990s [10]. This idea was compatible with the so-called macrophage theory of depression that was inspired by the effects of cytokine administration on the mood of healthy volunteers and the positive association between inflammatory diseases, such as rheumatoid arthritis and depression [11]. Subsequent research has shown that circulating cytokines are increased in clinically depressed patients [12], and that cytokine therapy in cancer and hepatitis increases the risk of developing clinical depression [13]. These findings have led to the hypothesis that pro-inflammatory cytokines are involved in the development of maladaptive behavioral and emotional changes characteristic of clinical depression [14]. Interestingly, when searching for inflammation and depression as title words of articles reporting human research on Pubmed, the category of major, bipolar or melancholic depression now yields the most hits (Table 1). Indeed, this category contains more articles than those of i.) coronary disease and myocardial infarction, ii) obesity, diabetes and metabolic syndrome, iii) kidney disease, iv) cancer, v) arthritis and rheumatoid conditions, vii) bowel conditions or vii) hepatitis. Hence, the role of inflammation in major depression is now more investigated than that of inflammation in depression related to a medical condition.

Table based on Pubmed search using the string (inflammation [TI] AND depression [TI]) NOT review NOT comment NOT “spreading depression” NOT (animal OR rat OR mouse) on 31/01/19. Nine-teen articles were excluded from the 153 obtained, 17 because articles did not report original findings and 2 because the term depression was used in a different context. The remaining 134 articles were classified according to the main somatic condition or mental disorder of interest in the article.

### 1.2. Immunopsychiatry and Psychoneuroimmunology

After this short historical overview of the links between inflammation and depression, the question arises as to what is new in the field of immunopsychiatry. This question also needs to be considered against the background in which the field of psychoneuroimmunology has been “studying the communication between the brain and the immune system” for already more than three decades [2]. It has been proposed that psychoneuroimmunology and immunopsychiatry “represent two different conceptualization of the brain-immune communication” [2] (p. 197). Psychoneuroimmunology in this view then puts “an emphasis on the notion that psychological and neural phenomena can influence the immune system” [2] (p. 197). Thus, it has been “argue[d] that the recent use of the term immunopsychiatry represents a hierarchical shift: It suggests that our brain no longer governs the immune system, but, on the contrary, that our behaviors and emotions are governed by peripheral immune mechanisms” [2] (p. 197). 

It is certainly true that psychoneuroimmunology initially addressed the question of whether the state of mind can influence the body’s immune system. However, as outlined above, the idea that activation of the innate immune systems can give rise to changes in behavior was already formulated in 1988. From the 1990s onwards, and as acknowledged by Reference [2] (p. 198), the mechanisms underlying sickness behavior have been progressively unraveled to constitute the second pillar of psychoneuroimmunology. Hence, the shift in focus or hierarchy in the bidirectional communication between the immune system and the brain can be considered to have occurred within psychoneuroimmunology rather than with the recent expansion of immunopsychiatry. 

The field of immunopsychiatry is therefore probably more dependent on psychoneuroimmunology then some of its proponents seem to believe. Epistemic dependence in science has been proposed to be the consequence of the “causal complexity” of the world [15] (p. 217) and the fact that “no individual scientist has the cognitive resources to oversee all the epistemically relevant aspects of the research projects that she—or: her team or community—is engaged in” [16] (p. 46). With this in mind, and given that psychoneuroimmunology has first come up with the idea that inflammation can give rise to depression, that immunopsychiatry now proposes to further address, the notion of epistemic dependence may be useful to describe the relationship between immunopsychiatry and psychoneuroimmunology. 

## 2. Inflammation and Stress as Accommodating “Umbrella” Concepts

### 2.1. Inflammation

Historically, inflammation has been associated with the four, now considered cardinal, signs of local inflammation described by Celcus in the first century, namely heat, pain, redness and swelling. In the mid-19th century, Virchow added disturbance or loss of function as a fifth sign and linked it to cellular pathology. At the end of that century, Metchnikoff, however, based on the observation of phagocytosis, emphasized the beneficial role of this cellular response for host defense and tissue homeostasis [17]. Thus, the concept of inflammation came to cover an entire range of responses spanning the normal and pathological.

During the 20th century, attempts were undertaken to distinguish types of inflammation with one important criterion being time. The acute phase response refers to the immediate and early reactions to tissue infection, trauma or injury. These responses are initiated by tissue macrophages and mast cells producing the cytokines Interleukin-1 (IL-1) and Tumor Necrosis Factor (TNF), which can act locally and distally and have pleiotropic activities [18]. In local endothelial cells, these cytokines and low-molecular-weight inflammatory mediators, such as arachidonic acid metabolites, induce expression of adhesion molecules, vasodilation and vascular leakage. When these cytokines, along with Interleukin-6 (IL-6), are released from the tissue where the initial infection or injury occurred into the blood stream, they can give rise systemic reactions like fever and the hepatic synthesis of acute phase proteins, such as C-reactive protein (CRP) and serum amyloid A [18]. 

Thus, another important criterion is the place or distance relative to the site of infection or injury and to distinguish local and systemic inflammatory responses. The term Systemic inflammatory response syndrome (SIRS) was coined in 1992 to qualify a condition when two of the following occurred: (1) A body temperature greater than 38 °C or less than 36 °C; (2) a heart rate greater than 90 beats per minute; (3) tachypnea, manifested by a respiratory rate greater than 20 breaths per minute, or hyperventilation, as indicated by a PaCO_2_ of less than 32 mm Hg; and (4) an alteration in the white blood cell count, such as a count greater than 12,000/mm^3^, account less than 4000/mm^3^, or the presence of more than 10 percent immature neutrophils [19,20]. Over the past three decades, it has become clear that pro-inflammatory cytokines play an important role in bringing about systemic inflammatory symptoms mediated by the central nervous system, such as fever, but also sickness behavior, in response to the detection of bacterial fragments [21]. 

However, inflammation also plays a broader role in tissue repair after infection or injury and in the “removal-replacement” of cells that show signs of cellular stress or malfunctioning and is considered to contribute to the restoration of tissue homeostasis and maintenance of organ function [17,22,23,24]. These responses may be qualified as low-grade inflammation or parainflammation and can be distinguished from the full-blown or high-grade inflammatory responses to tissues infection and injury. 

Finally, it is important to acknowledge that local inflammatory responses depend on the tissue in which they occur. For example, in response to injection of the same amount of bacterial lipopolysaccharide (LPS) fragments, the skin displays the full blown local inflammatory response, whereas the parenchyma of the central nervous system show delayed cellular infiltration with minimal recruitment of neutrophils [25]. Some hallmarks reminiscent of peripheral inflammation can, however, occur in the brain parenchyma after severe CNS injuries, such as stroke. This so-called neuroinflammation has been proposed to be characterized by increased cytokine expression, activation of microglia, immune-cell recruitment and neurodegenerative tissue damage [26]. But over the years, the term neuroinflammation has been employed rather loosely after the observations of just microglial activation or increased cytokine expression in the brain in response to peripheral and psychological stressors [26]. 

### 2.2. Stress, Depression and Inflammation

Repeated or chronic psychological stress has long been considered a risk factor for the development of clinical depression and to give rise to so-called depression-like behaviors in animals [27,28,29]. Interestingly, clinical depression has, in a subset of patients, been reported to be accompanied by changes in immune responses, including a rise in the number of circulating leukocytes, lymphopenia [30,31,32], altered lymphocyte responses to mitogens [32,33,34,35], decreased neutrophil phagocytosis, but augmented monocyte phagocytosis [35,36] and increased circulating concentrations of IL-6 and the acute phase protein CRP [37,38,39]. This led several authors at the end of the 1990s to put forward the idea that pro-inflammatory cytokines are causal factors in depression while acknowledging that stress and depression had classically been associated with reduced immune responses [35,40,41,42]. However, a couple of years later, a critical review considering measures of leucocyte trafficking and lymphocyte subsets, leucocyte functioning, and markers of immune activation in major depression concluded that “there are many inconsistent and even contradictory findings” [43].

The term stress is an example of an ‘umbrella concept’ that has proven useful because it allowed for (1) encompassing divers conditions (hemorrhage, restraint, etc.) based on their ability to induce non-specific neuroendocrine responses, and in particular activation of the Hypothalamo-Pituitary-Adrenal (HPA)-axis resulting in the release of glucocorticoids, and (2) studying the role of these neuroendocrine responses in health and disease. Once the general concept was formulated, different categories of stress could be distinguished based on the eliciting stimuli or conditions between systemic, homeostatic or physiological stressors and neurogenic, emotional or psychological stressors. Interestingly, homeostatic-physiological and emotional-psychological stressors activate different brain structures and circuits, yet both activate the paraventricular nucleus of the hypothalamus that regulates the HPA-axis [44,45].

Although infection is considered a homeostatic-physiological stressor and thus links stress and inflammation, a relationship between emotional-psychological stress and inflammation has also been put forward more recently. Psychosocial stress is well-known to be associated with increased incidence of disease [46], but the classic acute stress-responsive HPA-axis is not consistently activated in chronic stress [47]. Based on genome-wide expression studies of circulating monocytes showing blunted glucocorticoid signaling but increased expression of transcripts with response elements for NF-κB, a pro-inflammatory transcription factor [48], inflammation has recently been put forward as a mediator between psychosocial stress and disease. 

It has been proposed that psychosocial stress-associated “chronic low grade inflammation needs to be carefully distinguished from responses to acute infectious stimuli” in that the former is (1) “systemic and not limited to a local site of injury or infection”, (2) of “lower magnitude than … inflammation that accompanies acute infection or sepsis”, (3) typically a longer term phenomenon, as opposed to the transient nature of inflammatory responses to infection”, and (4) not due to an “apparent stimulus, such as infection or injury” [49] (p. 181). It is also important to point out that the vast majority of studies have, in the absence of an apparent local stimulus, focused on circulating mediators, such as cytokines and acute phase response proteins. Thus, increases in plasma IL-6 and to a lesser extent CRP have been found in elderly individuals taking care of a spouse with a chronic medical condition, persons with a low socioeconomic, victims of childhood abuse or maltreatment and patients with depression in comparison to respective controls [49]. In addition, acute laboratory psychosocial stress tasks, including the Trier social stress and the Stroop tests, have also been shown to be followed by increases in circulating IL-6 [49]. Finally, a recent meta-analysis of the effects of acute psychological stress in humans concludes that it reliably increases circulating IL-6, IL-1β, IL-10 and TNFα, but not CRP [50].

Among the mechanisms that have been proposed to underlie psychological stress-associated low-grade systemic inflammation, increased adrenergic signaling as a result of higher sympathetic nervous system activity has been put forward, since catecholamine action can activate pro-inflammatory intracellular NF-κB signaling and augment the number of circulating cells with inflammatory activity [49]. Interestingly, animal studies have shown that the prototypical uncontrollable stress conditions of immobilization and inescapable foot shock increase plasma IL-6 and splenic IL-1 via catecholamine action [51,52]. However, and although many laboratory stressors in humans and animals give rise to, and clinical depression is associated with, increases in circulating catecholamines, differences in catecholamine response between clinical depression and uncontrollable stress in animals have been observed [53,54]. It is important to point out here that subjecting animals to acute stressors is considered less relevant to clinical depression than exposing them to chronic stress regimens, such as chronic mild stress or repeated social defeat. Importantly, chronic unpredictable mild stress also increases serum IL-6, IL-1β, IL-10 and TNFα [55] while repeated social defeat stress results in higher circulating IL-6 concentrations [56,57]. 

Taken together, the available evidence indicates that acute psychological stressors, likely through catecholaminergic signaling, as well as chronic stress and depression can give rise to increased circulating concentrations of IL-6, which is often presented as a marker of systemic inflammation. However, very few of these studies have mentioned alternative interpretations. Indeed, increases in circulating IL-6 or CRP could well be related to cell “removal-replacement”, malfunctioning or stress, which have been proposed as triggers of low-grade inflammation or parainflammation at the tissue level [17,22,23,24]. It thus remains to be established whether or not psychological stress causes low-grade inflammation or if the effects are limited to the increases in circulating IL-6 or CRP concentrations.

### 2.3. Experimental Psychological Stress and Neuroinflammation?

In addition to raising circulating IL-6 concentrations, immobilization stress in rodents has been shown to increase IL-1 production in the brain [58,59]. Restraint, inescapable foot shock, forced swim and chronic social defeat stress have also been shown to result in increased IL-1β synthesis in the brain, even though often the increase only occurs under some experimental circumstances [60,61,62]. Moreover, in the case of inescapable footshock, this stress-induced rise in brain IL-1β mRNA has been shown to be accompanied by increased expression of the LPS-recognizing molecule CD14, which may be interpreted to reflect activation of microglia [63]. In a systematic review of the effects of psychological stress on the microglial specific marker ionized calcium binding adaptor molecule 1 (Iba-1), it was concluded that there is good evidence that psychosocial stressors, including chronic foot shock, restraint and social defeat in adult rodents lead to elevated microglial activity in the brain [64]. Furthermore, chronic unpredictable stress results in depressive-like behaviors, as well as changes in microglia morphology and function, of which some can be attenuated by cerebral overexpression of the IL-1 receptor antagonist (IL-1ra) or knockdown of Colony Stimulating Factor 1 (CSF-1) [65,66]. In addition to microglial activation, chronic social defeat has also been reported to enhance recruitment of mononuclear immune cells to the brain perivascular spaces [67] and to result in the breakdown of the blood-brain barrier [68]. However, other groups have shown that microglial phagocytic capacity was increased after chronic social defeat in the absence of changes in microglial morphology and infiltration of mononuclear immune cells [69]. These apparent discrepancies may be related to differences in stressor intensity, as well as to regional differences as chronic exposure of rats to cat odor results in both microglial activation and immune cell infiltration in the area postrema, an area where the blood-brain barrier is non-functional [70]. 

Altogether the available data suggest that features of neuroinflammation, such as increased CNS pro-inflammatory cytokine expression, glial activation, brain recruitment of immune cells and breakdown of the blood-brain barrier, occur after some acute and chronic stress conditions in rodents, including chronic social defeat. However, there is some tendency in the field to conclude as to the occurrence of neuroinflammation when only one single feature of neuroinflammation, for example microglial activation, has been observed [64].

## 3. Attempts to Mark Inflammation and Depression

A biomarker can be defined as “a characteristic that is objectively measured and evaluated as an indicator of normal biological processes, pathogenic processes, or pharmacologic responses to a therapeutic intervention” [71] (p. 91), “a laboratory measurement that reflects the activity of a disease process” [72] (p. 189) or as “specific biological features, or any substance, structure, or process that can be measured in the body to aid in distinguishing the presence or absence of a specific disease” [73,74] (66 p. 464). Well known biomarkers are serum low-density lipoprotein (LDL) cholesterol for cardiovascular disease and prostate specific antigen (PSA) for prostate cancer.

Many biomarkers have been proposed and tested in the context of sepsis, which has long been considered a condition characterized by high-grade systemic inflammation. Although C-reactive protein (CRP) has been proposed as a marker for sepsis, its specificity for this condition is actually low [75,76,77]. The possibility that CRP could instead constitute a general clinical biomarker of the non-specific acute phase and inflammatory response to injury and infection has been raised, even though the available evidence often does not allow CRP to be considered that way [75,78,79]. Importantly, differences between human CRP and CRPs in other animals exist in terms of its behavior as an acute phase protein, thus rendering the comparison between human and animal studies far from straightforward [78]. In addition, and relevant for the human literature, it has also been pointed out (1) that different CRP isoforms may have opposite effects on inflammation; (2) that elevated circulating CRP may reflect (para)inflammation restoring tissue homeostasis and (3) that psychological resources interact with socioeconomic status in men to predict CRP levels [22,80,81]. Hence, increased circulating CRP concentrations cannot be simply interpreted as reflecting inflammation.

Cytokines, and in particular IL-6, rapidly increase during severe systemic inflammation, but its specificity for sepsis as a single biomarker, just like for CRP, turns out to be low [77,82]. Instead, and like CRP, IL-6 has been proposed as a general marker of inflammation [83,84]. However, it is also likely (1) that increased systemic concentrations of IL-6 reflect local low-grade or (para)inflammation restoring tissue homeostasis; (2) that IL-6 bound to soluble receptors and acting on the membrane bound transducer molecule gp130 results in pro-inflammatory effects whereas free IL-6 binding to membrane IL-6 receptors associated with gp130 gives rise to anti-inflammatory effects; and (3) that psychological resources along with socioeconomic status also determine IL-6 levels, at least in men [22,80,85,86,87,88]. Therefore, increased plasma concentrations of IL-6 cannot always be considered to mirror inflammation and may, in some cases, even reflect ongoing anti-inflammatory responses.

Increased brain binding of Positron-Emission Tomography (PET) ligands with affinity for Translator protein (TSPO) has been proposed as a biomarker of microglial activation and neuroinflammation after it was shown that TSPO is mainly expressed in microglia and that TSPO binding is increased after traumatic brain injury and in neurodegenerative diseases [89,90]. While an initial report on a rather small sample did not observe differences in TSPO [91], later studies, including more patients indicated increased TSPO binding in forebrain structures in medication-free patients with major depression compared to healthy controls [92,93,94]. However, the interpretation of increased TSPO binding as a biomarker of microglial activation or neuroinflammation has been criticized. Indeed, a recent paper points out that it is highly likely “that the pathological meanings of altered TSPO binding or expression are disease-specific” and that “altered TSPO binding or expression … may reflect other pathophysiological processes, such as abnormalities in cell metabolism, energy production and oxidative stress….” [95].

Putative biomarkers of the transition between chronic stress and major depressive disorders have been proposed that are related to the HPA axis, such as the ratio of mineralo- and glucocorticoid receptor expression and activation, the balance between the sympathetic and parasympathetic nervous systems through heart rate variability measures, and gut function and dysbiosis [96]. Interestingly, a meta-analysis indicates that plasma concentrations of CRP and IL-6 have a significant, albeit small, association with the development of depressive symptoms over time [97]. However, based on the above discussion pointing out the difficulties in considering IL-6 and CRP markers of inflammation, it cannot be concluded from the association of IL-6 and CRP with the evolution of depressive symptoms that this implies that inflammation accompanies their development [98].

Several studies have measured cytokine concentrations in the cerebrospinal fluid (CSF) of depressed patients. The first among these reported higher CSF concentrations of IL-1beta, but lower concentrations of IL-6 and no change in TNFα in depressed patients compared to controls [99]. Intriguingly, later studies found either no difference in CSF IL-6 [100] or increased CSF IL-6 concentrations between patients with depression [101,102,103]. In addition, these studies show that no correlation exists between blood and CSF IL-6, but that CSF concentrations were higher than those in serum, suggesting “that CSF IL-6 is of central origin” [101,102]. In contrast, blood and CSF CRP correlate in depressed patients, but with CSF CRP concentrations being lower than those measured in the circulation [104]. Even if the results are mixed, it may be the case that in a subcategory of depressed patients CSF IL-6 concentrations are elevated as the result of increased IL-6 production.

## 4. Antidepressants and Anti-Inflammatory Drugs May not be “Surgical” Intervention Tools

Therefore, if, as outlined above, CRP and IL-6 cannot be simply considered biomarkers of inflammation, then the association of CRP and IL-6 with the development of depressive symptoms cannot be interpreted to provide evidence for a link between inflammation and depression that is not due to a medical condition. This conclusion gives rise to the question of what type of scientific approaches, other than association studies, could be relevant to address one of the core hypotheses of immunopyschiatry that inflammation plays a causal role in depression. 

Pharmaceutical drugs have long been considered as tools for intervention in establishing causal relationships in the life sciences. It is therefore of interest to consider the effects of anti-inflammatory drugs on symptoms of depression and those of anti-depressant drugs on signs of inflammation as evidence relevant for the hypothesis that inflammation causes depression, even in the absence of a medical condition. Moreover, and in addition to the role of pharmaceutical drugs in basic science, there is currently a great deal of interest in medicine and the pharmaceutical industry to determine if already existing drugs have beneficial effects beyond the conditions for which they are currently prescribed. The potential for anti-inflammatory drugs to have antidepressant effects is part of the evidence put forward by proponents of immunopsychiatry: “Randomized controlled studies using anti-inflammatories for depression have shown therapeutic effects [105]” [2] (p. 198). Finally, structural similarities between antidepressants affecting serotonin signaling (agomelatine, duloxetine and vortioxetine) and non-steroidal anti-inflammatory drugs (NSAIDs: Diclofenac, ketoprofen, naproxen and nabumetone) have recently been pointed out [106]. This thus would provide a structural basis for the claims that NSAIDS have antidepressant effects and antidepressants anti-inflammatory effects. 

Several authors have recently considered the available data and concluded that serotonin-reuptake inhibitor antidepressants appear to have anti-inflammatory effects, but that NSAIDs do not seem to possess antidepressant effects. Indeed, serotonin-reuptake inhibitors have been shown to not only inhibit in vitro pro-inflammatory cytokine production by immunocompetent cells after stimulation with, for example, bacterial LPS, but also to lower plasma TNFα, but not IL-6, concentrations in depressed patients that responded to treatment with these drugs [107,108]. Meta-analyses indicate beneficial effects of polyunsaturated fatty acids, which are known to be anti-inflammatory, on major depression when given in conjunction with antidepressants [105] However, reviews of the efficacy of non-steroidal anti-inflammatory drugs (NSAIDs) in depression point out that a randomized clinical trial did not show any significant effect and that only one of several retrospective cohort studies concluded that NSAIDs improve depressive symptoms [109,110,111]. 

Yet several groups point out that the effects of anti-inflammatory or immune-modulatory drugs should be evaluated in the subset of patients with depression showing increased CRP levels, which, as indicated above, is often interpreted to reflect inflammation [111,112]. It will therefore be interesting to follow the outcome of an ongoing randomized controlled trial addressing the effects of a single administration of single-dose of a humanized monoclonal antibody against the IL-6 receptor in patients with depression and serum concentrations of CRP ≥ 3 mg/L [113]. Nevertheless, given that IL-6 cannot be considered to simply reflect inflammation as different signaling pathways are likely to mediate pro- and anti-inflammatory effects, this trial may not constitute the critical test for the central hypothesis of immunopsychiatry that inflammation plays a role in major depression. 

## 5. Thinking Fast Regarding Inflammation and Depression

In logic, an inference is the process of reasoning in which a conclusion is derived from observations or premises. Induction is a type of inference in which a general conclusion is based on a limited number of observations (for example, concluding that swans are white based on observations of several hundred white swans across different parks) and is often used in science and medicine. For the establishment of a marker for a certain condition or for the evaluation of the effect of an intervention on an outcome, science and medicine therefore often employ statistics to determine the likelihood with which the pattern observed in a limited sample could be due to chance.

### 5.1. Insufficiently-Met Criteria-Based Inferences

Besides the general problem of induction according to which the general conclusion will always be underdetermined regardless of the number of observations, avoidable ‘underdetermined’ inferences occur when a conclusion is drawn in spite of the fact that all the required criteria to reach that conclusion have not been met. Diagnostic criteria in medicine often include the presence of one or several obligatory main criteria and several optional secondary criteria of which some should be met as well. For example, according to the Rome-IV criteria in order to diagnose Irritable Bowel Syndrome, recurrent abdominal pain has to be present on average at least 1 day/week over 3 months and needs to be associated with two of the following: Pain on defecation, a change in the frequency of stool, or a change in the form (appearance) of stool [114]. Hence, a physician diagnosing Irritable Bowel Syndrome after examining a patient who reports abdominal pain every other day or so for half a year when defecating but without a change in stool frequency or aspect would engage in an insufficiently-met criteria-based inference. 

### 5.2. Reverse Inferences

In other circumstances, when one encounters evidence for an event B that occurred consequent to an initial event A and an induction seems safe, one may be led to presume that A has occurred the next time B is encountered. This kind of reasoning has been coined ‘reverse inference’ or ‘affirming the consequent’ and constitutes an important heuristic strategy in science and medicine, but is also prone to risks. Reverse inferences are widespread in science and medicine simply because they can generate new hypotheses to be tested. However, the question of whether or not a reverse inference can be accepted, and if so under what conditions, is debated in different domains of science and medicine. 

One of the general matters at stake in these debates is whether a phenomenon of interest can be brought about in one or several ways. A recent debate on the interpretation of brain images when it comes to pain may be informative here. Pain is defined as an “unpleasant sensory and emotional experience associated with actual or potential tissue damage” (https://www.iasp-pain.org) and is considered to be “determined by a specific pattern of neural activity at the cortical level”, which can be studied by functional brain imaging [115] (p. 212). Functional brain imaging can identify statistical dependencies between experimental variables of interest related to the perception of pain and measured parameters reflecting brain activity. However, the probability of correctly predicting the occurrence of the perception of pain based on the presence of a pattern of brain activity (the reverse inference) depends on how many other conditions result in the same brain activity [115]. Proponents of the idea that one can predict pain from patterns of brain activity with machine learning have pointed out that “ruling out potential other explanations [for the pattern of brain activity] is an open-ended challenge, requiring many tests across studies, but ultimately an empirical one” [116] (p. 639). The question then becomes whether or not a “pain-signature” of brain activity can be validated and, if so, when, depending on the number of alternative explanations that have been considered.

When it comes to the link between inflammation and depression, it seems that part of the biomedical community has moved beyond the now widely-accepted assertion that medical conditions characterized by inflammation come with a higher risk of developing depression to postulate the occurrence of inflammation when a depressed individual is encountered. While this reverse inference, which seems to be one of the main claims of immunopsychiatry, is possible and should not be dismissed beforehand, a few points may exist where the aggregated evidence in favor of this claim may be less solid due to alternative explanations. And contrary to the possibility of predicting pain from a pattern of brain activity, no systematic machine learning strategies seem to have been proposed to date to address reverse inferences when it comes to the link between inflammation and depression. 

### 5.3. Potential Insufficiently-Met Criteria-Based and Reverse Inferences in Immunopsychiatry

Therefore, one of the heuristic reverse inferences of immunopsychiatry is to postulate, based on the now widely accepted idea that inflammation can lead to depression, that when an individual is diagnosed with major depression inflammation, should be suspected. However, it is important to consider the evidence that has been put forward so far to support this claim as it may include insufficiently met criteria-based inferences and reverse inferences. 

To illustrate the occurrence of insufficiently-met criteria-based inferences in immunopsychiatry, inflammation is a good starting point. The diagnosis of local inflammation is the typical conclusion when a physician observes the four cardinal signs swelling, heat, redness and pain. But since pain, for example, can occur in the absence of injury and prior to inflammation in case of injury or infection, it is important to not rely only on pain as a single symptom when suspecting local inflammation. Similarly, to conclude as to the presence of a systemic inflammatory response syndromes (SIRS) requires a minimum of two out the following four criteria to be met: Temperature > 38 °C or < 36 °C, heart rate > 90/min, respiratory rate > 20/min or PaCO2 < 32 mm Hg (4.3 kPa) and white blood cell count >12,000/mm^3^ or < 4000/mm^3^ or >10% immature bands. Hence, just a body temperature above 38°C is not sufficient to be qualified as a SIRS, which makes sense because an elevated body temperature alone may well be due to heat shock. Although the criteria for systemic inflammation have been widely used in clinical practice for more than 25 years, no consensus conference has yet endorsed criteria for low-grade systemic inflammation or parainflammation, which are the constructs used in immunopsychiatry. Similarly, no widely-accepted criteria exist for clinical practice regarding the presence of neuroinflammation. However, and as outlined above, clear criteria have been proposed to qualify a condition as neuroinflammatory [26]. For the authors of the proposed criteria: “[I]t is becoming increasingly clear that all four signs of inflammation—increased cytokines, activated microglia, T-cell recruitment and neurodegenerative tissue damage—should be assessed before applying the label of neuroinflammation to any neurological or psychiatric disorder” [26] (p. 624). Therefore, just an increase in brain pro-inflammatory cytokine production or a sign of glial activation is not sufficient to conclude as to the occurrence of neuroinflammation in a mental disorder or animal model thereof.

To be diagnosed with depression according to the DSM symptoms must include “a prominent and persistent period of depressed mood or markedly diminished interest or pleasure in all, or almost all, activities”, plus four additional symptoms for at least two weeks, among which are psychomotor slowing and loss of weight. However, proposed animal models of depression or assessment of depressive-like symptoms in animals often only mimic or address one feature of these DSM criteria. Hence, models of and studying symptoms of depression in animals may expose one to insufficiently-met criteria-based inferences. 

Biomarkers are interesting cases when it comes to criteria-based and reverse inferences. The common feature of the various descriptions of biomarkers is that they constitute measurable indicators of a disease process or response to treatment. Their objective nature makes biomarker somewhat akin to signs of disease obtained with a physical examination. However, the conclusions that can be drawn from the measurement of biomarkers will depend on the place that they have been attributed among diagnostic criteria, for example based on consensus conferences. Hence, coming to a diagnosis solely based on measurement of a biomarker often exposes one to engage in an insufficiently met criteria-based inference. 

As pointed out above, the proposed biomarkers CRP and IL-6 turn out to lack specificity for sepsis, which has long been considered as the prototypical systemic inflammatory condition. They may, however, constitute markers for artery disease [117,118,119]; but [120,121] that is known to be associated with inflammation. But this does not imply that CRP and IL-6 should be considered non-specific markers of inflammation. Indeed, no formal validation of CRP or IL-6 as a general biomarker of inflammation seems to exist to date. Instead, the reasoning often seems to be as follows: “Any condition that causes release of interleukin (IL) 6 and other cytokines triggers the synthesis of CRP and fibrinogen by the liver. Thus, CRP level can be used a measure of ongoing inflammation and cell necrosis, and levels are particularly elevated in states …, such as sepsis…” [122] (p. 139). Moreover, and as discussed above, alternative explanations may exist for increased circulating CRP or IL-6 concentrations. Finally, it has been proposed that “C-reactive protein elevation can be caused by conditions other than inflammation and may reflect biologic aging” [123] (p. 535). Consequently, basing any diagnosis of a disease involving inflammation solely on increased CRP or IL-6 levels has currently to be considered as an insufficiently-met criteria-based inference. 

Furthermore, biomarkers may also become prone to types of reverse inferences when they are considered beyond the use or conditions for which they have been validated. This would be the case when increased levels of a biomarker, validated to be part of the diagnostic criteria for a disease, are considered to reflect or even be part of potential disease-underlying processes or mechanisms, for example inflammation. The risk for such conclusions is probably even higher for constructs, like low-grade systemic inflammation, for which no widely-accepted diagnostic criteria have been established to date. These kinds of interference are frequently made in studies assessing CRP and IL-6 in relationship to stress or depression and that conclude as to the role of inflammation in these conditions. Here the critical point is not so much that increases in CRP or IL-6 would not be validated as biomarkers or sufficient to reach the diagnosis of a disease in which inflammation occurs, but that the very idea of a marker as potentially indicating a disease seems to have been replaced by one according to which elevated CRP or IL-6 means or reflects (low-grade) systemic inflammation. 

Finally, when it comes to medical drugs, physicians are supposed to determine which medication they will prescribe to a patient based on a full diagnosis. However, in the absence of a definitive diagnosis based on symptoms and signs, reverse inferences based on the effects of drugs are frequently made. For example, a physician or a patient may reason that one must have suffered from depression or inflammation if one is feeling better after having taken an antidepressant or an anti-inflammatory drug. Not only does this occur frequently in medical practice, but it also seems to underlie the predictive validation criterion for animal models of depression, namely the ability to predict in an animal the clinical effects of a treatment [124,125]. However, drugs often have other effects than the ones they were initially selected and marketed for. Therefore, concluding that an individual had depression solely on the basis of a drug that has antidepressant effects improving that individual’s condition is a kind of reverse inference that is often not warranted.

## 6. Conclusions

The aims of the present review were to provide some historical background and to consider the evidence presented in favor of the claim that inflammation is causally involved in major depression. While this claim is one of the central pillars of the emerging field of immunopsychiatry, links between inflammation and depression have previously been addressed by the field of psychoneuroimmunology. Instead of a proposed division of labor in which psychoneuroimmunology would study top-down mental-to-brain-to-immune phenomena and immunopsychiatry bottom-up immune-to-brain-to-mental effects [2], immunopsychiatry can be considered as a field of translational research in which hypotheses generated by basic science are tested in a clinical context. Immunopsychiatry then seems to specifically address the hypothesis that inflammation causes depression in the absence of a medical condition after psychoneuroimmunology has identified mechanisms that may underlie depression due to a medical condition. But in doing so immunopsychiatry will continue to be partly dependent on psychoneuroimmunology. 

Given that insufficiently-met criteria-based and reverse inferences seem to be operating to some extent in the field, immunopsychiatry is encouraged to reach out to different fields of basic and clinical science to specify concepts and constructs, as well as to consider potential alternative interpretations of and explanations for findings obtained. Indeed, replacing the presently used insufficiently validated biomarkers with clear definitions of low-grade systemic inflammation will help the field mature. With regard to biomarkers it is also hoped that the combination of different potential biomarkers will prove helpful to better characterize low-grade system inflammation and neuroinflammation, as well as to distinguish between the two, for example by measuring IL-6 and immune cells in both the systemic blood circulation and cerebrospinal fluid. In addition, and just as has been suggested for the emerging field of microbiome-gut-brain research (Birk, Brain Behav. Sci., in press), immunopsychiatry would also benefit from better specifying the types of stress it considers. Coming to clearer operational definitions of types of inflammation and stress can, in turn, be expected to favor translational research between the clinic and basic science. 

Clearer definitions and specification of subtypes rather than using poorly-defined accommodating ‘umbrella’ concepts could also indicate alternative interpretations of and explanations for phenomena. For example, an increase in IL-6 or CRP alone does not necessarily imply that inflammation is ongoing, but may be part of tissue repair or anti-inflammatory responses. Also, it is not because a drug has antidepressant or anti-inflammatory properties that all of its effects necessarily indicate that depression or inflammation was ongoing. It is safer to interpret its effects in terms of biochemical structure or function and acknowledge all of its potential sites of actions, for example as a serotonin-reuptake inhibitor that may affect serotonin signaling both in the brain and body of animals. Finally, inflammation or increases in potential biomarkers of inflammation should not necessarily be treated as a potential cause of depression and could even be a consequence. Interestingly, major depression has long been known to be associated with increased circulating noradrenalin concentrations [54,126]. Moreover, increased noradrenaline concentrations have, under some circumstances, been shown to stimulate pro-inflammatory cytokine expression and the pro-inflammatory NF-κB intracellular signaling pathway [127,128]. It seems therefore possible that part of the inflammation proposed to cause major depression in immunopsychiatry could be the consequence of changes in catecholamine concentrations.

It is likely that some of the critical points developed here regarding immunopsychiatry’s claim that inflammation is a causal factor in depression not due to a medical condition apply more generally to postulates of emerging fields in science and medicine. But the hope for immunopsychiatry is that pointing out some of the potential problems will allow for a clearer picture of its current strengths and limitations and help the field mature.

## Figures and Tables

**Table 1:** Number of Pubmed articles reporting original research with inflammation and depression in their titles and classified according to somatic conditions or mental disorders.

Condition or Disorder	Nb.	References (Authors, Journal, Year)
Major, bipolar or melancholic depression and their treatments	25	Panizzutti et al., Acta Neuropsychiatr., 2018; Mehta et al., Brain Behav. Immun., 2018; Felger et al., Mol. Psychiatry, 2018; Crawford et al., Hum. Mol. Genet., 2018; Kruse et al., J. Clin. Psychiatry, 2018; Faugere et al., J. Affect. Disord., 2018; Holmes et al., Biol. Psychiatry, 2018; Niemegeers et al., Neuropsychobiology, 2016; Lindqvist et al., Psychoneuroendocrinology, 2017; van Dooren et al., Brain Behav. Immun., 2016; Haroon et al., Mol. Psychiatry, 2016; Felger et al., Mol. Psychiatry, 2016; Al-Hakeim et al., J. Affect. Disord., 2015; Weinberger et al., Brain Behav. Immun., 2015; Grosse et al., Brain Behav. Immun., 2015; Tsai et al., Bipolar Disord., 2014; Krogh et al., Brain Behav. Immun., 2014; Rawdin et al., Brain Behav. Immun., 2013; Lamers et al., Mol. Psychiatry, 2013; Vogelzangs et al., Transl. Psychiatry, 2012; Maes et al., J. Affect. Disord., 2012; Maes et al., Prog. Neuropsychopharmacol. Biol. Psychiatry, 2012; Wolkowitz et al., PloS One, 2011; Shelton et al., Mol. Psychiatry, 2011; Wong et al., Mol. Psychiatry, 2008
Ageing, elderly	16	Rozing et al., Psychoneuroendocrinology, 2019; Niles et al., Psychoneuroendocrinology, 2018; Johnson et al., Int. Psychogeriatr., 2017; Das, Biodemography Soc. Biol. 2017; Gallagher et al., Int. J. Geriatr. Psychiatry, 2017; Theeke et al., Open J. Nurs., 2016; Mezuk et al., PloS One, 2016; Lai et al., Nutr. Res., 2016; Brown et al., J. Gerontol. A Biol. Sci. Med. Sci., 2016; Arts et al., J. Am. Geriatr. Soc., 2015; Hiles et al., J. Psychiatr. Res., 2015; Lu et al., Respir. Res., 2013; Stewart et al., Brain Behav. Immun., 2009; Davidson et al., Am. J. Cardiol., 2009; Pizzi et al., Eur. Heart J., 2008; Kop et al., Am. J. Cardiol., 2002
Coronary disease or myocardial infarction and their treatments	16	Mommersteeg et al., Brain Behav. Immun., 2016; Ma et al., J. Cardiovasc. Pharmacol., 2016; Xiong et al., Psychosom. Med. 2015; Nikkheslat et al., Brain Behav. Immun., 2015; Williams et al., Psychosomatics, 2014; Steptoe et al., Brain Behav. Immun., 2013; Munk et al., Int. J. Cardiol., 2012; Kupper et al., J. Affect. Disord., 2012; Bot et al., J. Psychosom. Res., 2011; Vaccarino et al., J. Am. Coll. Cardiol., 2007; Whooley et al., Biol. Psychiatry, 2007; Buriachkovskaia et al., Ter. Arkh., 2006; Janszky et al., Biol. Psychiatry, 2007; Toker et al., J. Occup. Health Psychol., 2005; Janszky et al., Brain Behav. Immun., 2005
Obesity, diabetes or metabolic syndrome and their treatments	9	Oriolo et al., Brain Behav. Immun., 2019; Murdock et al., Stress Health, 2018; Shenhar-Tsarfaty et al., Mol. Med., 2016; Rethorst et al., J. Clin; Psychiatry, 2014; Lamers et al., Mol. Psychiatry, 2013; Silić et al., J. Affect. Disord., 2012; Olszanecka-Glinianowicz et al., Mediators Inflamm., 2009; Emery et al., Obes. Surg., 2007; Benson et al., Brain Behav. Immun., 2008
Kidney disease and dialysis	8	Gencer et al., J. Am. Coll. Nutr.. 2018; Nowak et al., Int. Urol. Nephrol., 2013; Choi et al., Nephron Clin. Pract., 2012; Li et al., Int. Urol. Nephrol.. 2011; Ko et al., Nephron Clin. Pract., 2010; Ibrahim and Salamony, Am. J. Nephrol., 2008; Kalender et al., Int. J. Clin. Pract., 2007; Kalender et al., Nephron Clin. Pract., 2006
Cancer and its treatments	6	Jacobs et al., J Psychosom Res. 2017; Rodrigues et al., Am. J. Hosp. Palliat. Care, 2016; Han et al., Psychooncology, 2016; Castro et al., Blood Purif., 2014; J. Affect. Disord., 2012; Bower et al., J. Clin. Oncol., 2011
Arthritis or rheumatoid conditions	3	Reddy et al., Psychopharmacol. Bull., 2018; Kojima et al., Arthritis Care Res. (Hoboken), 2014; Kojima et al., Arthritis Rheum., 2009
Bowel conditions and their treatments	2	Gorrepati et al., Int. J. Colorectal Dis., 2018; Jizhong et al., Gastroenterol. Res. Pract., 2016
Hepatitis and its treatment	2	Hepgu et al., Neuropsychopharmacology, 2016; Felger et al., Physiol Behav. 2016
